# Semi-Metric Topology of the Human Connectome: Sensitivity and Specificity to Autism and Major Depressive Disorder

**DOI:** 10.1371/journal.pone.0136388

**Published:** 2015-08-26

**Authors:** Tiago Simas, Shayanti Chattopadhyay, Cindy Hagan, Prantik Kundu, Ameera Patel, Rosemary Holt, Dorothea Floris, Julia Graham, Cinly Ooi, Roger Tait, Michael Spencer, Simon Baron-Cohen, Barbara Sahakian, Ed Bullmore, Ian Goodyer, John Suckling

**Affiliations:** 1 Department of Psychiatry, University of Cambridge, Cambridge, United Kingdom; 2 Cambridge and Peterborough Foundation NHS Trust, Cambridge, United Kingdom; 3 MRC/Wellcome Trust Behavioural and Clinical Neuroscience Institute, University of Cambridge, Cambridge, United Kingdom; 4 Department of Radiology, Icahn School of Medicine at Mount Sinai, New York, New York, United States of America; 5 Department of Psychology, Columbia University, New York, New York, United States of America; 6 Department of Psychiatry, University of Oxford, Medical Sciences Division, Oxford, United Kingdom; Universiteit Gent, BELGIUM

## Abstract

**Introduction:**

The human functional connectome is a graphical representation, consisting of nodes connected by edges, of the inter-relationships of blood oxygenation-level dependent (BOLD) time-series measured by MRI from regions encompassing the cerebral cortices and, often, the cerebellum. Semi-metric analysis of the weighted, undirected connectome distinguishes an edge as either direct (metric), such that there is no alternative path that is accumulatively stronger, or indirect (semi-metric), where one or more alternative paths exist that have greater strength than the direct edge. The sensitivity and specificity of this method of analysis is illustrated by two case-control analyses with independent, matched groups of adolescents with autism spectrum conditions (ASC) and major depressive disorder (MDD).

**Results:**

Significance differences in the global percentage of semi-metric edges was observed in both groups, with increases in ASC and decreases in MDD relative to controls. Furthermore, MDD was associated with regional differences in left frontal and temporal lobes, the right limbic system and cerebellum. In contrast, ASC had a broadly increased percentage of semi-metric edges with a more generalised distribution of effects and some areas of reduction. In summary, MDD was characterised by localised, large reductions in the percentage of semi-metric edges, whilst ASC is characterised by more generalised, subtle increases. These differences were corroborated in greater detail by inspection of the semi-metric backbone for each group; that is, the sub-graph of semi-metric edges present in >90% of participants, and by nodal degree differences in the semi-metric connectome.

**Conclusion:**

These encouraging results, in what we believe is the first application of semi-metric analysis to neuroimaging data, raise confidence in the methodology as potentially capable of detection and characterisation of a range of neurodevelopmental and psychiatric disorders.

## Introduction

Complex networks, introduced some fifteen years ago [1, 2] have found applications in many areas of science and technology [3–7]. The introduction of network analysis to neuroimaging investigations has broadened interpretations from primarily compartmentalized models of brain regions responding to external stimuli, to distributed models where the key elements are the connections between regions, both in the presence and absence of cognitive load.

Networks can represent many scales of the brain: from neural interactions to inter-regional connectivity. At large-scales, a connectivity network of the brain—the connectome—may be constructed that reflects anatomical connections, functional (correlational) connections, or effective (influential) connections [8].

Functional connectivity networks have enjoyed the most exposure as they are relatively easy to construct and are not dependent on strong, prior neurobiological hypotheses. Typically, Pearson’s correlation is used to capture synchronously activated regions, although other metrics may give different perspectives; for example, spectral mutual information measures the strength of associations between regions, which is related to feedback causality [9, 10]. In general, it is important to recognize that properties of the connectome are not invariant to the variables that are the foundations for its construction.

Many graph theoretical measures can be derived from both binary (where connections or either present or absent between any pair of nodes in the graph) and weighted networks where, in our case, the edges represent the degree of synchronicity of brain activation. Real-world weighted networks, including the human brain connectome, have a high number of transitivity violations [11–15]. Descriptively, a transitivity violation occurs if the distance of an indirect path between two nodes is less than the distance of the direct path between them. This type of network is called *semi-metric* and is embedded in a non-metric topology [15]. Generally, any weighted network will have some degree of semi-metricity. In recent work, we have shown that in many types of real-world networks the levels of semi-metricity are high [11, 13–15]. In other words, networks have a high degree of redundancy or increased sharing of information amongst communities.

In this paper we begin by formally introducing the concept of semi-metricity and then undertake an analysis of this type on functional human brain networks derived from resting-state blood oxygenation-level dependent (BOLD) sensitive MRI obtained from groups of adolescents with autism spectrum condition (ASC), moderate-to-severe major depressive disorder (MDD), and matched control participants. ASC is a developmental disorder with origins in the genome, in utero and early life environments. The phenotype includes reduced social interaction and communication and is apparent from an early age and has an approximately 4.3:1 sex-ratio in favour of boys [16]. MDD is an affective disorder that often emerges in adolescence when the sex-ratio of its prevalence changes from 1:1 to 3:1 in favour of girls pre- and post-puberty respectively. Genes play a role in the vulnerability of individuals to MDD, but environmental experiences during childhood are significant risk factors [17].

MDD and ASC reside at opposite extremes on a factor meta-analysis across a wide spectrum of mental health disorders [18], thus making them ideal to test both the sensitivity and specificity to psychopathologies of semi-metric network analysis. Moreover, from the neuroimaging literature ideas have evolved on both ASC [19] and MDD [20] in terms of functional connectivity, and what role aberrant changes in communication may have on the phenotype. Here, we explore how semi-metric analysis of functional connectivity in brain networks cross-sectionally characterizes ASC and MDD, and if they offer a new perspective on established neurobiological models.

Functional connectivity from the BOLD signal has generally been studied in low frequencies, below 0.1Hz, although recent studies have pointed to the importance of higher frequencies in the range 0.1–0.25Hz [21, 22]. Wavelet multi-scale analysis was used in this study to decompose the time-series and connectomes derived from the correlation of wavelet coefficients in four contiguous frequency bands (scales) ranging from 0.03–0.25Hz [23]. Initially the global levels of semi-metricity were calculated for each group from connectomes with nodes defined by regions of an anatomical atlas. Subsequently, the locations and nature of the observed differences were investigated. Areas where there was an overabundance (relative to controls) of metric (direct) connections were interpreted as *constrained* information processing. Conversely, where semi-metric (indirect) connections were relatively more prevalent, the involvement of additional regions was interpreted as *dispersed* communication reflective of the co-activity of multiple regions. Subsequently, the degree distribution of the semi-metric and metric connections of ASC and MDD participants were compared to control participants using a node disruption index.

The most prevalent model of the psychopathology of MDD, at least in adults, is over-activation of the limbic system in response to negatively valenced stimuli coupled to under-activation of brain circuits involved in cognitive control, primarily the lateral prefrontal cortex [24]. Thus, it might be assumed that connections in these regions might well be aberrant. A frequently cited model for characterising the connectome of individuals with ASC is that of local over-connectivity relative to reduced long-range connectivity [25]. Subsequent evidence points to connectivity changes in posterior cingulate and insula cortex [19], what appears to be convergent is that there are effects are distributed throughout the brain. Thus, we hypothesise that changes in the ASC group would similarly be widely distributed. Although it is difficult to predict the specific direction of effects, differing directions of effects in ASC and MDD relative to control subjects were expected in terms of the proportion of connections that were semi-metric.

### Theoretical Background

Graph theory is the study of the mathematical relations and their properties commonly applied to complex networks [15, 26]. These include computer systems, transport infrastructure, social and ecological relationships, or neuronal structures in the central nervous system, in fact any set of interconnected objects that have a non-trivial organization.

The shortest path between nodes of a network is an important feature as it is a measure of information exchange between two or more nodes connected by edges with which there is an associated weighting. It is defined as the route between two nodes that minimizes the sum of the weights of the edges that are traversed. When the shortest path is directly between the nodes it is known as a *metric* edge. Conversely, when the shortest path is circuitous—via additional nodes—it is known as a *semi-metric* edge, [11–15].

Finding the shortest path has traditionally been a challenging optimization problem in computer science. One approach [15, 27] is to translate from a proximity space, where connections have their original weights, to a distance space where the connections now refer to the dissimilarity between nodes.

A high value of proximity between two nodes in a graph can be closely related to one another, but it is also possible that the two nodes can have higher proximity by transitivity with the introduction of a common, third (or more) node(s). To find the shortest path the proximity can be maximized through transitive closure, where the simple relations between nodes (for example, correlations) are converted to relations that describe all the destinations that are reachable from a given node.

More formally, proximity graphs are a class of weighted graphs which are symmetric, reflexive and bounded on the unit interval [0, 1], but are not transitive. Any weighted graph bounded in a non-normalized interval [*a*,*b*], where *a*, *b* are real numbers, can be normalized by a unique linear function onto the unit interval [28, 29].

To build up a more intuitive understanding of transitivity in weighted graphs, we converted our proximity graphs, *P*, to distance graphs via an isomorphism, *φ* One possibility for the proximity-to-distance conversion function is [28, 29]:

$$\varphi :d_{ij}=\frac{1}{w_{ij}}-1$$

A distance graph *D*, obtained via *φ* from *P*, does not in general yield a metric topology. This is because for a pair of nodes *i* and *j*, the triangle inequality may be violated: *d*_*ij*_ ≥ *d*_*ik*_ + *d*_*kj*_ for some element, *k*. Thus, the shortest path between two elements may not be the direct edge, but rather an indirect path via a number of edges. Clearly, semi-metric behaviour is a question of degree. For some pairs of nodes in a distance graph there are many indirect paths that violate the triangle inequality. Distance functions that violate the triangle inequality are referred to as *semi-metric* [30].

To measure the degree of semi-metric behaviour in a graph we have previously introduced the *semi-metric ratio* [31, 32]:

$$s_{ij}\;=\;\frac{d_{ij}}{{\overset{´}{d}}_{ij}}$$

where ${\overset{´}{d}}_{ij}$ is the shortest, direct or indirect, distance between *i* and *j* in distance graph *D* calculated by metric closure, or equivalently by Johnson’s algorithm [33].*s*_*ij*_ is positive and > 1 for semi-metric edges, and = 1 for metric edges. *s*_*ij*_ is only applied to semi-metric edges *d*_*ij*_ where $0<{\overset{´}{d}}_{ij}<d_{ij}$ (see [15] for more details).

An interpretation of a semi-metric path in isomorphic proximity space in a connectome is if two regions (nodes) are semi-metrically connected, they synchronously co-activate along with all the regions in the semi-metric path. In other words, there is a *dispersion* of communicability across regions. Complementarily, metric connections occur without the significant involvement of any other regions; that is, the information exchange is *constrained* between the two nodes.

### Connectome semi-metric and metric percentages

**Fig 1:**
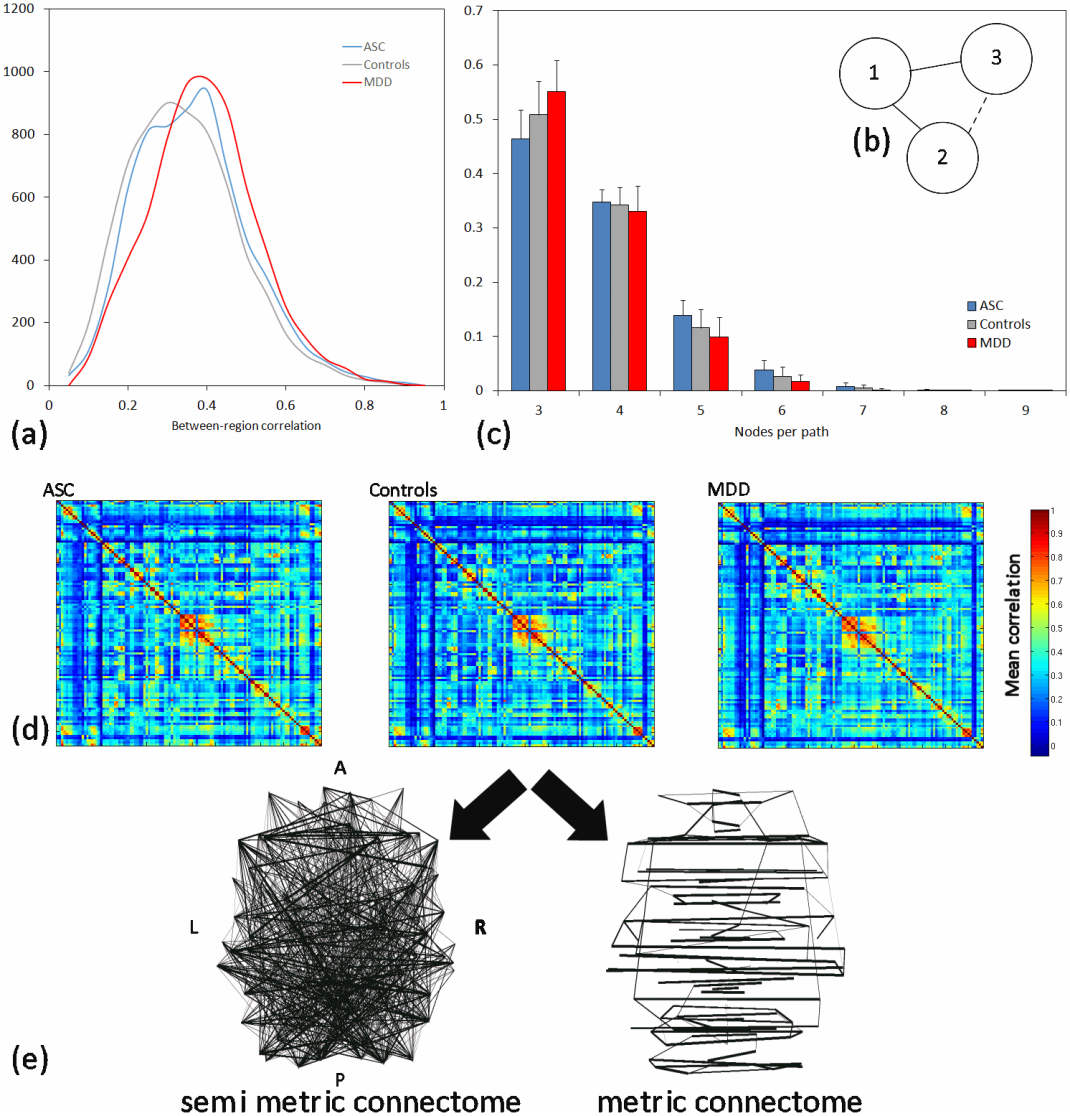
Overview of network properties and analysis. (a) Histogram of correlation coefficients (i.e. edge weights) for each group. (b) Schematic diagram of a simple network with a semi-metric connection between nodes 1 and 2 (dashed edge) due to a shorter indirect path comprising edges between nodes 2 and 3 and 3 and 1 (solid edges). (c) The distribution of number of edges for semi-metric paths for each group. (d) Proximity matrices averaged across participants, for each group. (e) Axial projections of metric and semi-metric backbones for the control group. The thickness of the edges represents the percentage of participants within each group with a semi-metric edge at that location, with percentages > 90% omitted.

The *semi-metric percentage (SMP)* measures the overall level of semi-metric behaviour of a network. This measure is obtained from the *semi-metric ratio*:

$$SMP\;=\;\frac{\sum_{i,j}\left(s_{ij}>1\right)}{\left|E\right|}$$

where |E| is the total number of connections in the network under consideration; that is, in proximity space, edges greater than zero. This measure represents the level of dispersal of communication in a functional brain network as semi-metrically correlated regions co-activate with the regions along the semi-metric path (Fig 1b).

*The metric percentage (MP)* measures are again obtained from the *semi-metric ratio*:

$$MP\;=\;\frac{\sum_{i,j}\left(s_{ij}\;=\;1\right)}{\left|E\right|}$$

The metric percentage measures the level of *constraint* in a functional brain network. Two regions are metrically correlated in that they co-activate without significantly co-activating other regions. The *MP* and *SMP* are complementary; that is, *SMP* = 1 –*MP*.

## Materials and Methods

### Participants

A sample of 35 healthy adolescents, 33 adolescents with ASC, and 35 adolescents with a DSM-IV diagnosis of MDD were selected for the study from a larger cohort by matching age and gender (see S1 Text). Demographic details are given in Table 1.

**Table 1:** Demographic characteristics of the participants.

Characteristics	ASC participants	MDD participants	Control participants
Gender (F/M)	11/22	18/17	19/16
Age range (years)	13.65–17.61	13.65–17.61	12.14–17.29
Age ± SD (years)	15.45 ± 1.39	14.92 ± 1.70	15.33 ± 1.39

SD = standard deviation

The ASC participants were recruited from local support groups, schools and the Autism Research Centre (University of Cambridge) database as part of the Cambridge Family Study of Autism. Participants were assessed with the Autism Diagnostic Schedule generic (ADOS-D) and the Autism diagnostic interview revised (ADI-R) to confirm a DSM-IV diagnosis of high functioning Autism or Asperger syndrome. No comorbidities were reported for these participants, and none were receiving prescribed medication. The typically developing controls group for this sample were recruited from local schools and community groups and had no family history of ASC. Details of this cohort have been described in detail [34].

The MDD participants were recruited as part of the MR-IMPACT study [35]. Participants were assessed according to the DSM-IV diagnosis as having moderate-to-severe MDD. Two participants with MDD had a comorbid diagnosis of psychosis for which they were prescribed risperidone. These two participants along with 14 others from the MDD group (i.e. 16 in total) were currently administered with selective serotonin reuptake inhibitors. Details are given in S1 Table. The comparison group of typically developing adolescents with no family history of depression were recruited from local schools by advertisement.

Written informed consent was given from a parent or legal carer and all participants gave their written assent to be involved, or written consent if they were over 16 years. This study was approved by the Cambridgeshire 1 Research Ethics Committee (Cambridge Family Study of Autism: 08/H0304/126), and the Cambridgeshire 2 Research Ethics Committee (MR-IMPACT: 09/H0308/168).

### MRI acquisition

Participants with ASC (n = 33) and 18 controls were scanned using a Siemens 3T Tim Trio scanner (Siemens Healthcare, Erlangen, Germany) located at the Medical Research Council Cognition and Brain Sciences Unit, Cambridge, UK. BOLD sensitive functional images were acquired with a gradient echo planar imaging sequence with the following parameters: repetition time = 2000ms, echo time = 30ms, voxel size = 3x3x3mm^3^, field of view = 192 x 192 mm^2^, 64 x 64 acquisition matrix and a 78° flip angle. In all, 32 slices were acquired descending in the transverse plane (slice thickness = 3 mm, slice gap = 25%) and 260 three-dimensional volumes acquired.

Participants with a diagnosis of MDD (n = 35) and 17 controls were scanned with an identical Siemens 3T Tim Trio scanner located at the Wolfson Brain Imaging Centre, University of Cambridge, UK using the same gradient echo planar imaging sequence as described above.

Imaging data were acquired from all participants whilst in wakeful rest (i.e. without any applied stimulus) with eyes closed.

### Functional connectivity networks

Imaging data were pre-processed to account for head motion [36] (BrainWavelet Toolbox, www.brainwavelet.org). In brief, the first four volumes of each resting state data set were removed to eliminate the non-equilibrium effects of magnetization leaving 256 volumes for analysis. Preprocessing steps then included slice acquisition correction using 7th order Lagrange polynomial interpolation; rigid-body head movement correction to the first volume using 5th order polynomial interpolation to estimate the realignment parameters (3 displacements and 3 rotations); obliquity transform to the structural image followed by affine co-registration to the skull-stripped structural image using a gray matter mask; standard space transform to the MNI152 template in Talairach space; and spatial smoothing (6 mm full width at half maximum). Additional processing for the correction of head motion included: unsupervised time series despiking in the wavelet domain; confound signal regression of the 6 motion parameters estimated during rigid body head movement correction and their first order temporal derivatives and ventricular cerebrospinal fluid (CSF) signal; high pass frequency filtering above 0.02 Hz,; and spatial smoothing (6-mm full width at half maximum Gaussian kernel).

To test for between-group differences in head motion, independent t-tests (assuming unequal variances) were undertaken of mean DVARS, the average root mean square variance across all brain voxels of volume-to-volume difference in percent signal change, and the final relative to initial image displacement (translations and rotations about orthogonal axes). These metrics represent rapid and slow rates of head motion, respectively. For each individual the mean time-series was extracted from each of 116 anatomically parcellated regions (i.e. nodes) based on the Eickhoff-Ziles atlas (EZ116) [37]. The extracted BOLD signals were decomposed into four frequency bands by wavelet transform [38]: scale 1, 0.125–0.25Hz; scale 2, 0.06–0.125Hz; scale 3, 0.03–0.06Hz; scale 4, 0.02–0.03Hz. [23]. The strength of a connection between two nodes was the Pearson’s correlation coefficient of the wavelet coefficients and proximity matrices constructed from the positive correlations between all nodes.

Non-negatively weighted, undirected connectomes were derived and further analysis undertaken using Matlab (MathWorks, U.S.A) yielding metric and semi-metric connectomes defined by the corresponding edge type and from which the scalar SMPs were calculated for spatial regions.

### Global, hemispheric and lobular semi-metric percentages

**Fig 2:**
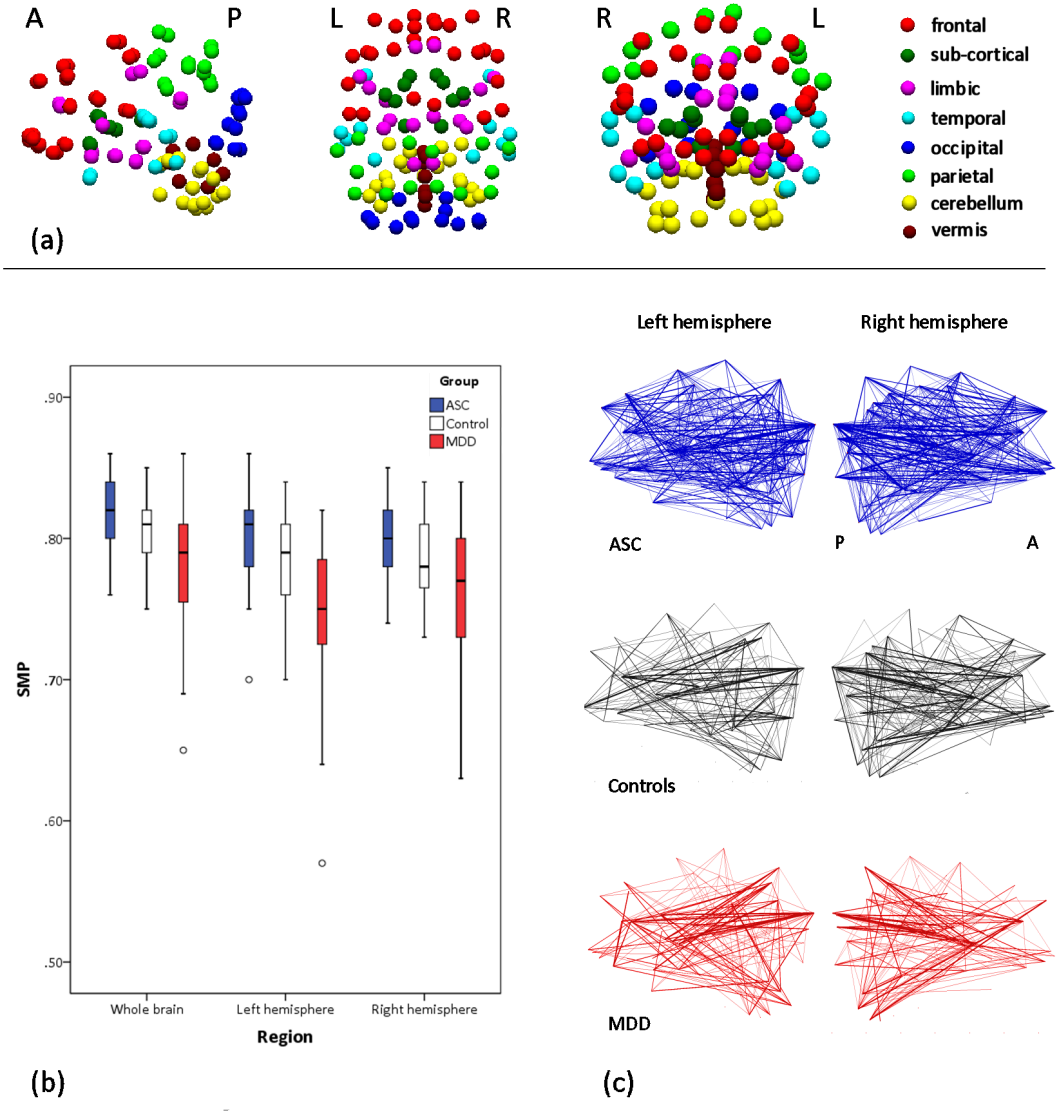
Semi-metric percentages and backbones. (a) Sagittal, axial and coronal projections of nodes coloured according to the modules in which they are contained. (b) Between-group comparisons (patient groups relative to controls) for whole brain, left and right hemisphere SMP displayed as box-and-whisker plots identifying the median by the central line, the 25^th^ and 75^th^ percentile ranges by the limits of the box, and the minimum and maximum range (excluding outliers) by the limits of the whiskers. Outliers are individually displayed and defined as values >1.5 the interquartile range from the 25% and 75% quartiles. (c) Sagittal projections of the left and right hemispheres of the semi-metric backbones for each group. The thickness of the edges represents the percentage of participants within each group with a semi-metric edge at that location, with percentages > 90% omitted.

To observe both the sensitivity and specificity of the semi-metric approach, group differences in the SMP were first tested at the whole-brain level, then a decomposition into sub-graphs for left and rights hemispheres, then decomposing hemispheric sub-graphs into lobes and the connections between them (defined by the Harvard-Oxford atlas, http://fsl.fmrib.ox.ac.uk/fsl/fsl4.0/fslview/atlas-descriptions.html, and colour coded in Fig 2a) to identify regions that contributed to any overall difference in SMP. These tests would not be independent, and thus a scheme was adopted whereby pursuance of statistical testing was predicated on the significance (i.e. p<0.05, two-tailed) of the test in the preceding level in the hierarchy. SMPs from between-lobe, within-hemisphere edges and between-hemisphere edges were tested as measures of more long-range connections.

### Within-group semi-metric backbones

Variation across individuals of the semi-metric connectomes was visually assessed by construction of the backbone for each group. An edge on the backbone is displayed with a thickness that denotes the percentage of individuals from the group that have a semi-metric connection at that location. The minimum percentage of individuals required to display an edge in the backbone is arbitrary, but was selected here at 90% for depiction of the key features whilst removing connections not commonly shared across the group and serve only to obscure the visualisation. Metric backbones may be constructed, but due to the smaller proportion of metric connections they are sparser and thus not as informative.

### Node degree and node disruption index

The degree of a node is the number of edges associated with that node. Trivially, for a fully-connected graph the degree of each node is identical. However, the semi-metric approach dichotomises edges as either metric or semi-metric, and so the degree for each node was then calculated for each connectome. The SMP is the proportion of edges that are semi-metric and is calculated within or between regions. The node degree indicates whether an excess of edges in the semi-metric connectome is preferentially localised to a few nodes or distributed more widely, in which case between-group differences in degree are less likely to be significant.

To summarise differences between groups in terms of their degree, the node disruption index [39], *K*, was calculated for metric and semi-metric connectomes separately. The between-group difference in mean degree was calculated for each node and plotted against the mean degree for that node in the control group. The index *K* was then estimated as the slope of the regression line. Values of *K* significantly less than 0 (i.e. p-value < 0.05 and 95% confidence interval not encompassing zero) indicate relative reorganisation of the connectome. The direction of the reorganisation depends on whether there is an excess of degree in patients or controls.

## Results

### Between-group comparisons of head motion

Between-group differences in mean DVARS were for ASC vs Controls: t(55.85) = 4.54, p = 3.01x10^-5^; and for MDD vs Controls: t(66.42) = -0.11, p = 0.92.

Between-group differences in the translations and rotations of the final volume relative to the initial volume were non-significant (p > 0.05) for both comparisons (Table 2).

**Table 2:** Comparison of motion parameters.

Comparison	Direction	t	df	p-value
ASC vs controls	x-displacement	0.79	49.24	0.43
	y-displacement	-1.08	61.36	0.28
	z-displacement	1.80	57.71	0.08
	Rotation about x	1.64	51.01	0.11
	Rotation about y	0.48	52.09	0.63
	Rotation about z	-1.96	50.15	0.06
MDD vs controls	x-displacement	1.07	57.84	0.29
	y-displacement	0.22	67.39	0.83
	z-displacement	1.32	63.37	0.19
	Rotation about x	-1.10	67.67	0.27
	Rotation about y	0.50	53.38	0.62
	Rotation about z	0.97	65.58	0.34

Between-group comparisons of the differences between final and initial image volumes of the translations and rotations around orthogonal axes (x, y, z) with an independent samples t-test assuming unequal variance, two-tailed.

df = degrees of freedom.

### Characterisation of metric and semi-metric connectomes

The histogram of correlation coefficients (Fig 1a) and mean proximity matrices (Fig 1d) for each group are similar in form and show no obvious qualitative differences. The number of negative Pearson correlation coefficients between regions was non-significant between the three groups (F(2,103) = 1.8392, p = 0.16). The distribution of the number of edges that make up the semi-metric paths across the whole brain is shown in Fig 1c, with again no differences between the groups. Axial projections of the connectomes (Fig 1e) for the control group demonstrate that metric connections tend to link homologous regions between hemispheres. Conversely, semi-metric paths primarily connect nodes within the hemispheres of the cerebrum and cerebellum.

### Between-group comparisons of semi-metric percentage

Between-group tests of SMP were conducted on connectomes derived from wavelet scales 2, 3 and 4. Scale 1 was discarded since it represents the frequency band in which uncorrelated noise from non-biological sources predominates [23]. Significant results in comparisons at the whole-brain level were obtained with scale 2 wavelet coefficients, representing the 0.06–0.12 Hz frequency range. There were no significant results at the whole-brain level with connectomes derived from wavelet scales 3 and 4 for either of group comparisons, but these results are included in supplementary information (S2, S3, S4 and S5 Tables) for completeness.

Significant between-group differences in SMP for the whole brain at wavelet scale 2 were observed in comparisons of both ASC and MDD groups with the control group, but with effects in opposing directions. Significant between-group differences were also observed in both left- and right-hemispheres again with opposing directions of effect; Fig 2b and Tables 3 and 4. Sagittal projections of the metric and semi-metric backbones are shown for each in group separately in Fig 2c. Semi-metric connections are generally sparser relative to controls (reduced SMP) for MDD participants, whilst connections are generally denser for ASC participants relative to controls (increased SMP).

**Table 3:** Semi-metric percentages for ASC vs control groups.

Region		Difference of means	Confidence Interval (95%)	p-value
Whole brain		0.015	0.002, 0.029	0.024*
Left hemisphere		0.018	0.001, 0.035	0.035*
Right hemisphere		0.016	0.001, 0.030	0.036*
Cerebellum		0.017	-0.007, 0.041	0.170
Vermis		0.030	-0.017, 0.077	0.208
Between-hemispheres		0.012	-0.002, 0.026	0.081
Left	Frontal	0.025	-0.002, 0.026	0.093
	Parietal	0.001	-0.004, 0.055	0.974
	Occipital	0.058	-0.052, 0.053	0.042*
	Temporal	0.042	0.002, 0.113	0.173
	Limbic	-0.014	-0.020, 0.103	0.560
	Subcortical	0.092	-0.060, 0.033	0.017*
	Betwen-lobe	0.018	0.017, 0.167	0.021*
Right	Frontal	0.022	-0.001, 0.046	0.059
	Parietal	-0.046	-0.095, 0.003	0.067
	Occipital	0.046	-0.016, 0.109	0.145
	Temporal	0.049	-0.006, 0.104	0.079
	Limbic	-0.028	-0.075, 0.020	0.246
	Subcortical	0.017	-0.055, 0.087	0.643
	Between-lobe	0.015	-0.001,0.028	0.032*

Regional comparison (two tailed t-test, df = 113) of semi-metric percentages for ASC vs control groups.

*p<0.05.

**Table 4:** Semi-metric percentages for MDD vs control groups.

Region		Difference of means	Confidence Interval (95%)	p-value
Whole brain		-0.027	-0.046, -0.009	0.005**
Left hemisphere		-0.035	-0.056, -0.013	0.002**
Right hemisphere		-0.026	-0.048, -0.005	0.017*
Cerebellum		-0.065	-0.096, -0.035	6.3x10^-5^ **
Vermis		-0.082	-0.138, -0.026	0.005**
Between-hemisphere		-0.025	-0.043, -0.007	0.007**
Left	Frontal	-0.048	-0.078, -0.017	0.003**
	Parietal	-0.016	-0.075, 0.042	0.582
	Occipital	-0.010	-0.072, 0.052	0.747
	Temporal	-0.076	-0.136, -0.016	0.014*
	Limbic	-0.031	-0.081, 0.020	0.228
	Subcortical	-0.018	-0.086, 0.050	0.604
	Between-lobe	-0.033	-0.052, -0.014	0.001**
Right	Frontal	-0.022	-0.053, 0.008	0.147
	Parietal	-0.045	-0.102, 0.012	0.111
	Occipital	-0.021	-0.090, 0.049	0.558
	Temporal	-0.036	-0.093, 0.020	0.204
	Limbic	-0.067	-0.117, -0.017	0.010**
	Subcortical	-0.048	-0.118, 0.022	0.178
	Between-lobe	-0.027	-0.046, -0.008	0.007**

Regional comparison (two tailed t-test, df = 113) of semi-metric percentages for MDD vs control groups.

*p<0.05,

**p<0.01.

Significant results at the whole-brain and hemispheric divisions of the cerebrum prompted a more detailed analysis of the modules (defined in Fig 2a) to identify contributory regions of between-group difference; Tables 3 and 4.

The ASC group had significant differences from controls in SMP (Table 3) in the left occipital and sub-cortical areas, with an increase in mean value and confidence intervals that do not overlap with negative (i.e. reduced) SMP values. The SMP associated with longer range connections between-modules in the left hemisphere were also significantly increased.

MDD participants were significantly different from controls in SMP (Table 4) in the left frontal and temporal lobes, right limbic system and cerebellum. Mean SMP was reduced with confidence intervals that did not encompassing positive (i.e. increased) SMP values. SMPs for between-hemisphere connections were significantly reduced as were between-module connections in both left and right hemispheres. A test of whole-brain SMP between unmedicated and medicated participants with MDD was non-significant (t(33) = 2.00, p = 0.054). Inspection of Tables 3 and 4 draws attention to the relatively larger magnitude of the mean SMP differences between MDD and control groups at the whole-brain level relative to the smaller mean differences between ASC and control groups. Furthermore, several p-values are at trend level (p < 0.1) for the ASC group at the modular spatial level, but non-significant regions for the MDD are all associated with p > 0.1. To summarise qualitatively, MDD is characterised by localised, large reductions in SMP whilst ASC is characterised by more generalised, subtle increases in SMP.

### Between-group comparisons of node degree

**Fig 3:**
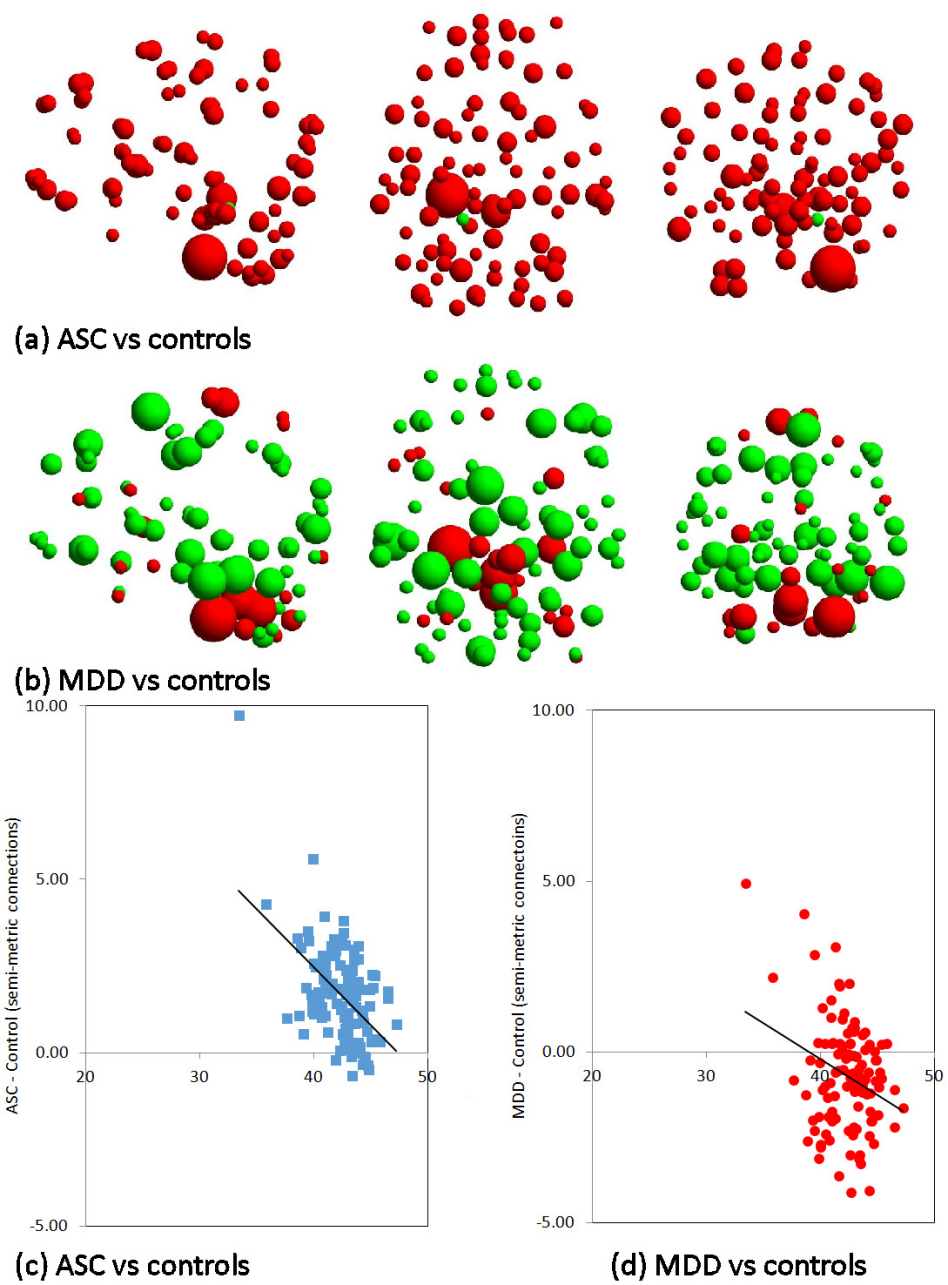
Node degree and node disruption indices. Sagittal, axial and coronal projections (left-to-right) of nodes for comparisons of node degree in the semi-metric network for each between-group comparisons: (a) ASC vs. controls; (b) MDD vs controls. The radius of the node is proportional to the average degree difference and the colour denotes the direction of the effect; red indicating increases and green decreases, relative to controls. Plots of the difference in mean degree between (c) ASC and (d) MDD, and controls against mean degree for controls, for the semi-metric network. Node disruption indices are defined as the slope of the regression lines, plotted on each graph.

Maps of mean degree difference between ASC (Fig 3a) and MDD (Fig 3b) groups compared to controls broadly recapitulate the results of SMP differences (Tables 3 and 4). ASC case-control differences in degree are mostly positive (Fig 3a) reflecting the spatially distributed nature that characterises the corresponding changes in SMP (Table 3). The node disruption index, *K* = -0.336 (Fig 3c: 95% CI -0.440 and -0.323; t(113) = 6.373, p = 4.15 x10^-9^) with almost all nodes showing an excess of connections in the ASC group (mean degree difference = 1.665 +/- 1.398). Nodes with the lowest degrees in the semi-metric connectome of controls have the greatest differences in ASC (Fig 3c).

In contrast to the results with ASC individuals, MDD degree differences with controls are approximately symmetrical around zero (mean degree difference = -0.655 +/- 1.625), with the largest differences in cerebellum and vermis and a trend towards larger changes in the right hemisphere (Fig 3b). The node disruption index, *K* = -0.209 (95% CI -0.341 and -0.077; t(113) = 3.147, p = 2.11 x 10^−3^). Thus, nodes with the lowest degrees in the semi-metric connectome of controls are generally more connected in MDD, and those with the highest degrees are the least connected (Fig 3b).

## Discussion

This study investigated the complex topological differences in brain networks in a cross-sectional examination of adolescents with ASC, MDD and a matched control group. This empirical framework allows us to test semi-metricity as a technique for the analysis of functional connectivity, and in particular whether it has the specificity and sensitive to developmental and psychiatric disorders that will make it useful to understanding the neurobiology of mental health and illness. Although previously applied to genomic analysis [40] there are, as far as we know, no published examples of its application to neuroimaging data. Nevertheless, the mathematical theory is well established [14, 15].

### Characterising the connectome with semi-metricity

Semi-metric analysis operates on undirected, weighted graphs. A connection is semi-metric if there is an indirect pathway that involves regions (network nodes) other than the original and target nodes that closes a transitivity (Fig 1b). In other words the correlation, representing the connection weight between these two regions, is “increased” in the proximity space by isomorphically summing correlations in the distance space between successive regions along the semi-metric path.

The distributions of correlation coefficients (Fig 1a) and the corresponding proximity (Fig 1d) matrices have similar forms. The metric and semi-metric connectomes derived identified metric connections as mainly between bilateral homologous regions and semi-metric connections as mainly intra-hemispheric (Fig 1e). In fact, these general observations were true for all three groups. Between-hemisphere, strong and symmetric connections have been reported and replicated with a variety of analytic techniques on numerous occasions previously [38, 41]. In this analysis these connections are characterised as metric and suggest a direct synchronicity that is largely independent of other regions. Conversely, semi-metric connections are intra-hemispheric and essentially local, with the majority of semi-metric pathways involving only 3 or 4 nodes (Fig 1b) which is in keeping with notions of high local efficiency in brain networks as an indicator of the information transfer in the vicinity of a node [42].

### Sensitivity and specificity of global SMP to MDD and ASC

Between-group tests of SMP were undertaken to assess the sensitivity of semi-metric analysis in comparing individuals with ASC and MDD to matched control participants, as well as the specificity via the comparative magnitude and direction of the effects.

Global (i.e. whole-brain) SMP was significantly different for both groups relative to controls (Tables 3 and 4; Fig 2a). Notably, relative to controls MDD was associated with a reduction in SMP whilst an opposite, increase was seen in ASC. These results indicate a clear sensitivity of SMP to these conditions and a relative specificity between them. It also points to a more general result in which there appears to be an optimum, or typical brain topography, and a deviation of SMP towards either more direct or indirect connections is associated with atypical function. The fact that SMP is able to discriminate between MDD and ASC at the coarsest scale (i.e. whole-brain) motivated refinement of the spatial components investigated—first hemispheres, then modules—to locate areas of greater or lesser contribution to the global effects.

### Characterisation of adolescents with ASC

ASC was associated with a broadly increased SMP. Specifically, significant increases in SMP were located in occipital, cerebellar and sub-cortical regions. These regions are concordant with recent data showing hyperconnectivity in the resting-state connectome in similar regions as well as widespread increases in functional connectivity between modules [25], similar to those detected here (Table 3). The broadly increased nodal degree also reflects the widespread nature of the differences with controls. Interestingly, nodes with the lowest degree in controls have the largest difference with the ASC sample (Fig 3c) suggesting that changes are in areas of more peripheral, less dispersed communication. Confirming this overall description, changes in key parameters of binary networks indicate a more random organisation of topology typified by a more distributed pattern and a reduction in connections through nodes characterised by their high degree (i.e. hubs) in brain locations associated with social and non-social cognitive functions [43].

The majority of the extant literature describing the functional connectivity of ASC comes from studies of brain networks under external stimulation that target specific cognitive processes. Whilst these studies suggest a decrease in connectivity associated with ASC, results demonstrating increased connectivity are not uncommon [44]. Looking more closely at this literature, it appears that methodological differences may play more than a minor role in determining the overall picture. In particular, techniques that introduce low-pass filtering to the preprocessing of the BOLD signal and that consider the entire brain tend to show increases in connectivity [44]. This view is supported by histological examination of post-mortem samples that have observed increased density of cortical minicolumns [45] and increased neuron number [46] in ASC. The semi-metric analysis encompassed BOLD sensitive data from the entire cortex and cerebellum and adopted a preprocessing strategy that included low-pass filtering by wavelet transform. Additionally, head movement artefacts that have been suggested to be the principal source of between-group difference [47] were specifically addressed in the development of the preprocessing pipeline [36]. However, there were significant between-group differences in mean DVARS, suggesting that rapid motion is remains issue in these individuals.

Interestingly, the number of neurons is reduced in the amygdala of those with ASC [48], counter to the general increase in cortical cell numbers noted above, but where significant changes in SMP were against the trend at larger spatial scales. Differences in the direction of the effect across many brain regions (Table 3) are symptomatic of the literature where variety is more common than convergence. Such heterogeneity of results has been attempted to be reconciled by suggesting that ASC is characterised by overconnectivity in brain systems associated with the phenotype, whilst underconnectivity is presented in those systems that are neurotypical, both probably as a result of aberrant developmental processes [44]. The results presented here suggest that it is the topology of the connectome that is locally variable, whilst the strengths of connections are universally increased.

The semi-metric percentage is not easily related to notions of over- or under-connectivity (i.e. the number or density of binary edges) as it is derived from the weighted connectome. Instead SMP is a topological property which, when increased relative to that in a control population indicates that information flow is preferentially routed circuitously—dispersed—and involves more regions that would otherwise be the case if the regions were metrically connected. On the face of it, this might appear to be a proxy for overconnectivity. However, the dichotomy between semi-metric and metric connections does not depend on the magnitudes of functional connectivity (here, positive values of Pearson’s correlation), but on the relative strengths of the paths involved.

An ancillary observation of differences in SMP in ASC is that they appear be more heterogeneous in the left hemisphere with several modules with significant effects, whilst in the right hemisphere, although SMP is significant overall, there are few individual loci of large effect size (Table 3). Typical brain development is characterised by left lateralisation of regions involved in language processing, and right lateralisation for regions sub-serving attentional demands [49, 50]. There is strong evidence from neuroimaging that both structure and function have reduced or reversed left lateralization in language regions in ASC [51, 52]. Additionally, decreases in serotonin synthesis are commonly observed in ASC with left cortical decreases being associated with greater language impairment [53]. The role of serotonin in brain development is well-known both in the growth of serotonergic neurons as well as regulating maturation of target brain areas [54]. Together, this is further evidence of the role of connectivity in the phenotype of ASC. Indeed, a large-scale study of resting state fMRI datasets archived in a public database demonstrated a loss of left-laterality in ASC involving Wernicke's and Broca’s areas [52]. Whether the SMP changes are part of the left-lateralization associated with ASC, or a more widely distributed imbalance in intra-hemispheric connectivity will require further investigation.

### Characterisation of adolescents with MDD

MDD in this age group is characterised by large decreases of intra-cerebral SMP located in both hemispheres (Table 4). Whilst significant reductions in SMP were located within frontal, temporal and cerebellar regions, as well as the connections linking modules and hemispheres, it is notable that the direction of the between-group effect of reduced SMP was consistent for all modules. In other words, the topology of functional connections is generally biased towards metric, direct connections. This is also visible from the corresponding semi-metric backbone where the number of edges at which 90% of the MDD patients had a semi-metric path is clearly reduced (Fig 2c).

Mean differences in node degree were approximately centred around zero suggesting that the semi-metric connectivity of many nodes is unchanged compared to controls, and that where differences do occur they are in the tails of the distribution (Fig 3d). In other words, regions of high, dispersed semi-metric connectivity in controls are deficient in connections, reflecting decreases in SMP in areas of the cerebral cortices (Table 4). However, nodes with lower connectivity in controls have an excess of connections in MDD, primarily in the cerebellum (Fig 3b). Here, the SMP is also significantly reduced implying that although the number of local, within-module semi-metric connections is reduced, the presence of longer-range edges to regions outside of the cerebellum is sufficient to cause an overall apparent increase in degree.

To our knowledge, the topology of the weighted functional connectome has not been previously studied in MDD. Indeed, there is also a paucity of literature on binarised connectomes in adolescent MDD. Notwithstanding this absence of prior evidence from the connectome, the regions identified are components of the fronto-limbic network linked with “bottom-up” processing of emotionally valent events and “top-down” cognitive control of emotion, an imbalance of which is postulated to underlie many of the common symptoms of depression [24, 55]. Seed-based connectivity analysis suggests an increase in coherence in the limbic system of adolescents with MDD [56]. The cerebellum is highlighted by a strong reduction in SMP, and is also known to have a role in emotional regulation [57]. What is common to these modules in this analysis is that the networks within and between them are characterised by a constraints in information flow; that is, they do not involve other regions in their synchronous activation.

In real world networks, information is usually passed via circuitous routes to allow nodes to condition or interpret information en route from source to destination. Bypassing these additional nodes, data potentially arrives in a form that is less compatible with that expected leading to difficulties in meaningful processing. Furthermore, information may be replicated or divided prior to transmission to then follow multiple routes and reassembled later [58]. An increase in metric connections may serve to diminish these possibilities. How such changes to network topology influences the behavioural phenotype of MDD awaits further study with a larger sample size, but a reduction in the complexity of information flow is suggestive of the pathophysiology that underlies rumination or the cognitive biases or difficulties in utilising new information that may predispose individuals to rumination or perseverate on prior non adaptive beliefs [59].

## Limitations

Functional connectivity remains a relatively new approach for neuroimaging. Some of the differences observed in psychiatric and developmental disorders using this technique have been suggested to arise, in part at least, from residual head motion following corrections typically applied to task-based fMRI [60, 61]. In this analysis there was a significant increase in mean DVARS in the ASC group relative to controls indicative of increased rapid head motion during data acquisition. The preprocessing pipeline used, including wavelet despiking has been shown to significantly ameliorate the effects of a wide variety of motion artefacts [36]. Nevertheless, motion cannot be ruled out as a source of between-group difference in SMP.

Another potential source of confound is the contribution of cardiac and respiratory signals to the BOLD-sensitive time-series upon which the SMPs are derived [62, 63]. In our experience the devices for measuring these signals within the confined space of the MRI scanner are found to be uncomfortable for those with psychiatric and developmental disorders, and particularly so in younger participants. Thus, to improve compliance and retention of participants we did not acquire these data. As a consequence, the contribution of their effects is unknown and the implicit assumption is made that they are equally distributed across individuals leading to no particular bias in any group.

Tests for differences in SMP within the control group as a result of scanning on two different, albeit identical MRI scanners, and between medicated and unmedicated MDD participants gave p-values close to significance (S1 Text). In general, inhomogeneity within groups serves to inflate the within-group variation leading to an increase in the likelihood of type II rather than type I errors. It may be that more homogeneous groups would give rise additional power to detect differences in SMP.

The large majority of functional connectivity studies under task-absent conditions have concentrated on frequencies <0.05Hz, usually selected through low-pass filtering of the BOLD-sensitive time-series. In this study, frequencies for analysis were selected through wavelet decomposition, which is particularly suitable by virtue of the 1/*f* power spectrum of these time-series in the frequency domain [42]. Several studies of psychiatric disorders using the wavelet decomposition method have preferentially reported results of the connectome in the range 0.06–0.125 Hz or similar [42, 64–67]. Between-group tests of SMP differences at wavelet scales corresponding to lower frequency bands were not significant at the whole-brain level (S2, S3, S4 and S5 Tables). This makes cross-referencing these results with the extant literature more problematic, and a degree of caution in this respect is therefore advisable.

## Conclusion

Semi-metric analysis of the human functional connectome constructed by inter-regional correlations between BOLD sensitive, low-frequency time-series acquired at rest, has both sensitivity and specificity to identify differences between MDD and ASC and a typically developing control group. The SMP denotes topological properties of the connectome that introduce ideas of complexity of information flow from region-to-region, and nodal degree indicates the regions that are involved in dispersed or direct processing.

The datasets used in this analysis were unbiasedly sampled from larger cohorts in a study that was primarily designed to test the technique of semi-metricity. MDD is characterised by increased metric connections in frontal and limbic regions that may underlie rumination which commonly accompanies depression. ASC in comparison has is predominantly characterised by a widespread increase in SMP, but with relatively smaller effect sizes.

These encouraging results motivate specific studies of MDD and ASC using the larger samples that are more representative of the respective populations, and exploring behavioural and cognitive relationships to semi-metricity as well as developmental correlates, both typical and abnormal. From this study, the clear discrimination of MDD and ASC relative to controls with effects that are aligned to previously reported differences in functional connectivity using other techniques raises confidence in semi-metricity as a methodology that is potentially capable of detection and accurate classification of a wide range of psychiatry and developmental disorders.

## Supporting Information

S1 Table Medication status of MDD group. (DOCX)

S2 Table Semi-metric percentages for ASC vs control groups at wavelet scale 3. (DOCX)

S3 Table Semi-metric percentages for MDD vs control groups at wavelet scale 3. (DOCX)

S4 Table Semi-metric percentages for ASC vs control groups at wavelet scale 4. (DOCX)

S5 Table Semi-metric percentages for MDD vs control groups at wavelet scale 4. (DOCX)

S1 Text Supplementary Information. (PDF)
