# Supplementary material for: Semi-Metric Topology of the Human Connectome: Sensitivity and Specificity to Autism and Major Depressive Disorder
Source: PLoS One. 2015 Aug 26;10(8):e0136388. doi: 10.1371/journal.pone.0136388 (PMC4550361; doi:10.1371/journal.pone.0136388)
Supplement: S1 Table — (DOCX) [file pone.0136388.s001.docx]

**S1 Table: Medication status of MDD group**

| Age (years) | Gender | Medication | Dose (daily) |
| --- | --- | --- | --- |
| 13.78 | female | fluoxetine | 40 mg |
| 16.96 | male | citalopram | 10 mg |
| 16.56 | male | fluoxetine  risperidone | 20 mg  1 mg |
| 14.45 | female | fluoxetine | 20 mg |
| 14.95 | male | fluoxetine | 20 mg |
| 13.88 | male | fluoxetine | 20/40 mg  (alternated) |
| 17.36 | female | fluoxetine | 20 mg |
| 17.44 | male | fluoxetine | 40 mg |
| 14.88 | male | fluoxetine | 20 mg |
| 16.67 | male | citalopram  risperidone | 40 mg  0.5 mg |
| 15.42 | male | fluoxetine | 10 mg |
| 17.52 | female | citalopram | 40 mg |
| 17.27 | female | fluoxetine | 10 mg |
| 13.65 | female | fluoxetine | 20 mg |
| 14.85 | male | fluoxetine | 10 mg |
| 15.43 | male | fluoxetine | 10 mg |

Age, gender and medication status at the time of MRI scanning for a sub-set of MDD participants.
