# Supplementary material for: Semi-Metric Topology of the Human Connectome: Sensitivity and Specificity to Autism and Major Depressive Disorder
Source: PLoS One. 2015 Aug 26;10(8):e0136388. doi: 10.1371/journal.pone.0136388 (PMC4550361; doi:10.1371/journal.pone.0136388)
Supplement: S2 Table — (DOCX) [file pone.0136388.s002.docx]

**S2 Table: Semi-metric percentages for ASC vs control groups at wavelet scale 3**

| Region | | Difference of means | Confidence Interval  (95%) | p-value |
| --- | --- | --- | --- | --- |
| Whole brain | | 0.005 | -0.005, 0.015 | 0.343 |
| Left hemisphere | | 0.009 | -0.004, 0.022 | 0.160 |
| Right hemisphere | | 0.004 | -0.010 , 0.018 | 0.614 |
| Cerebellum | | 0.011 | -0.014, 0.036 | 0.384 |
| Vermis | | 0.012 | -0.040, 0.065 | 0.647 |
| Between-hemispheres | | 0.007 | -0.004, 0.019 | 0.183 |
| Left | Frontal | 0.014 | -0.011, 0.039 | 0.262 |
|  | Parietal | 0.021 | -0.034, 0.076 | 0.452 |
|  | Occipital | 0.075 | 0.003, 0.147 | 0.042* |
|  | Temporal | 0.024 | -0.037, 0.085 | 0.436 |
|  | Limbic | -0.017 | -0.057, 0.023 | 0.394 |
|  | Subcortical | -0.022 | -0.099, 0.055 | 0.572 |
|  | Between-lobe | 0.006 | -0.006, 0.018 | 0.310 |
| Right | Frontal | 0.012 | -0.012, 0.036 | 0.309 |
|  | Parietal | 0.006 | -0.047, 0.060 | 0.809 |
|  | Occipital | 0.061 | -0.015, 0.136 | 0.114 |
|  | Temporal | 0.005 | -0.049, 0.059 | 0.851 |
|  | Limbic | -0.0005 | -0.046, 0.044 | 0.980 |
|  | Subcortical | -0.010 | -0.089, 0.069 | 0.807 |
|  | Between-lobe | 0.003 | -0.010, 0.016 | 0.662 |

Regional comparison (two tailed t-test, df =113) of semi-metric percentages for ASC vs control groups at wavelet scale 3.

*p<0.05.
