# Supplementary material for: Semi-Metric Topology of the Human Connectome: Sensitivity and Specificity to Autism and Major Depressive Disorder
Source: PLoS One. 2015 Aug 26;10(8):e0136388. doi: 10.1371/journal.pone.0136388 (PMC4550361; doi:10.1371/journal.pone.0136388)
Supplement: S3 Table — (DOCX) [file pone.0136388.s003.docx]

**S3 Table: Semi-metric percentages for MDD vs control groups at wavelet scale 3**

| Region | | Difference of means | Confidence Interval  (95%) | p-value |
| --- | --- | --- | --- | --- |
| Whole brain | | -0.008 | -0.021, 0.004 | 0.182 |
| Left hemisphere | | -0.011 | -0.026, 0.004 | 0.160 |
| Right hemisphere | | -0.011 | -0.026, 0.004 | 0.154 |
| Cerebellum | | -0.041 | -0.067, -0.016 | 0.002* |
| Vermis | | -0.055 | -0.115, 0.004 | 0.067 |
| Between-hemispheres | | -0.010 | -0.022, 0.002 | 0.096 |
| Left | Frontal | -0.010 | -0.035, 0.0135 | 0.384 |
|  | Parietal | -0.006 | -0.063, 0.051 | 0.828 |
|  | Occipital | 0.038 | -0.030, 0.107 | 0.271 |
|  | Temporal | -0.035 | -0.097, 0.027 | 0.260 |
|  | Limbic | -0.042 | -0.083, 0.001 | 0.050* |
|  | Subcortical | -0.062 | -0.137, 0.014 | 0.110 |
|  | Between-lobe | -0.012 | -0.026, 0.002 | 0.085 |
| Right | Frontal | -0.022 | -0.048, 0.003 | 0.081 |
|  | Parietal | 0.012 | 0.642, 0.630 | 0.660 |
|  | Occipital | 0.055 | -0.025, 0.135 | 0.174 |
|  | Temporal | 0.008 | -0.044, 0.061 | 0.748 |
|  | Limbic | -0.046 | -0.099, 0.008 | 0.094 |
|  | Subcortical | -0.046 | -0.129, 0.036 | 0.263 |
|  | Between-lobe | -0.011 | -0.025, 0.003 | 0.130 |

Regional comparison (two tailed t-test, df =113) of semi-metric percentages for MDD vs control groups at wavelet scale 3.

*p<0.05.
