# Supplementary material for: Semi-Metric Topology of the Human Connectome: Sensitivity and Specificity to Autism and Major Depressive Disorder
Source: PLoS One. 2015 Aug 26;10(8):e0136388. doi: 10.1371/journal.pone.0136388 (PMC4550361; doi:10.1371/journal.pone.0136388)
Supplement: S4 Table — (DOCX) [file pone.0136388.s004.docx]

**S4 Table: Semi-metric percentages for ASC vs control groups at wavelet scale 4**

| Region | | Difference of means | Confidence Interval  (95%) | p-value |
| --- | --- | --- | --- | --- |
| Whole brain | | 0.002 | -0.005, 0.009 | 0.603 |
| Left hemisphere | | 0.004 | -0.008, 0.015 | 0.543 |
| Right hemisphere | | -0.002 | -0.012, 0.008 | 0.710 |
| Cerebellum | | 0.012 | -0.012, 0.036 | 0.329 |
| Vermis | | 0.002 | -0.050, 0.054 | 0.949 |
| Between-hemispheres | | 0.005 | -0.004, 0.015 | 0.289 |
| Left | Frontal | 0.022 | -0.006, 0.050 | 0.117 |
|  | Parietal | 0.005 | -0.049, 0.060 | 0.845 |
|  | Occipital | 0.006 | -0.066, 0.079 | 0.862 |
|  | Temporal | -0.007 | -0.066, 0.052 | 0.813 |
|  | Limbic | -0.013 | -0.062, 0.036 | 0.596 |
|  | Subcortical | -0.014 | -0.098, 0.071 | 0.750 |
|  | Between-lobe | 0.001 | -0.010, 0.012 | 0.858 |
| Right | Frontal | -0.008 | -0.039, 0.022 | 0.588 |
|  | Parietal | -0.044 | -0.101, 0.014 | 0.132 |
|  | Occipital | 0.085 | 0.011, 0.160 | 0.026* |
|  | Temporal | 0.033 | -0.044, 0.109 | 0.396 |
|  | Limbic | -0.028 | -0.078, 0.023 | 0.278 |
|  | Subcortical | 0.022 | -0.052, 0.095 | 0.561 |
|  | Between-lobe | -0.002 | -0.011, 0.008 | 0.725 |

Regional comparison (two tailed t-test, df =113) of semi-metric percentages for ASC vs control groups at wavelet scale 4.

*p<0.05.
