# Supplementary material for: Semi-Metric Topology of the Human Connectome: Sensitivity and Specificity to Autism and Major Depressive Disorder
Source: PLoS One. 2015 Aug 26;10(8):e0136388. doi: 10.1371/journal.pone.0136388 (PMC4550361; doi:10.1371/journal.pone.0136388)
Supplement: S5 Table — (DOCX) [file pone.0136388.s005.docx]

**S5 Table: Semi-metric percentages for MDD vs control groups at wavelet scale 4**

| Region | | Difference of means | Confidence Interval  (95%) | p-value |
| --- | --- | --- | --- | --- |
| Whole brain | | -0.003 | -0.010, 0.004 | 0.398 |
| Left hemisphere | | -0.001 | -0.012, 0.010 | 0.889 |
| Right hemisphere | | -0.007 | -0.017, 0.003 | 0.170 |
| Cerebellum | | -0.034 | -0.062, -0.007 | 0.015* |
| Vermis | | -0.056 | -0.109, -0.002 | 0.041* |
| Between-hemispheres | | -0.009 | -0.017, 0.0001 | 0.054 |
| Left | Frontal | -0.001 | -0.031, 0.029 | 0.942 |
|  | Parietal | -0.006 | -0.058, 0.046 | 0.814 |
|  | Occipital | 0.008 | -0.056, 0.073 | 0.794 |
|  | Temporal | -0.048 | -0.113, 0.018 | 0.150 |
|  | Limbic | -0.021 | -0.066, 0.024 | 0.353 |
|  | Subcortical | 0.004 | -0.077, 0.084 | 0.926 |
|  | Between-lobe | -0.002 | -0.012, 0.009 | 0. 740 |
| Right | Frontal | -0.021 | -0.048, 0.006 | 0.126 |
|  | Parietal | 0.014 | -0.035, 0.064 | 0.568 |
|  | Occipital | 0.018 | -0.042, 0.078 | 0.560 |
|  | Temporal | 0.020 | -0.049, 0.089 | 0.566 |
|  | Limbic | -0.058 | -0.107, -0.010 | 0.020* |
|  | Subcortical | -0.004 | -0.084, 0.076 | 0.915 |
|  | Between-lobe | -0.008 | -0.017, 0.002 | 0.103 |

Regional comparison (two tailed t-test, df =113) of semi-metric percentages for MDD vs control groups at wavelet scale 4.

*p<0.05.
