# Supplementary material for: Semi-Metric Topology of the Human Connectome: Sensitivity and Specificity to Autism and Major Depressive Disorder
Source: PLoS One. 2015 Aug 26;10(8):e0136388. doi: 10.1371/journal.pone.0136388 (PMC4550361; doi:10.1371/journal.pone.0136388)
Supplement: S1 Text — (PDF) [file pone.0136388.s006.pdf]

S1 Text: Supplementary Information

The cohort used in this analysis is a combination of data from two studies: The Cambridge Family Study of Autism (CFSA. N = 56 ASC; N= 40 controls) and the Magnetic Resonance Imaging adjunct study to the Improving Mood with Psychoanalytic and Cognitive Therapies clinical trial (MR-IMPACT. N = 122 MDD; N = 39 controls). Selection of the cohort was drawn from the larger study samples matching for age and gender.

All resting functional imaging datasets were pre-processed for head motion [30] removing those datasets that failed this process (N = 2 MDD; N = 6 controls), had a high degree of head motion (N = 10 ASC; N = 3 MDD; N = 19 controls), signal dropout N = 1 MDD; N = 1 control), failed co-registration or did not have sufficient coverage of the brain for parcellation (N = 2 ASC; N = 3 MDD; N = 11 controls). Datasets from a further N = 6 ASC participants were removed due to the absence of demographic data.

The sample available from which to draw the cohort used in this analysis was thus, CFSA: N = 38 ASC and N= 36 controls; MR-IMPACT: N = 89 MDD; N = 30 controls). A systematic search was then conducted to obtain the best possible matching of ASC, MDD and control groups in terms of age and gender whilst maximising group sizes (ASC N = 33; MDD N = 35; controls N = 36). Additionally, the control group contained equal numbers of participants from each of the contributing studies (N = 18 CFSA + 18 MR-IMPACT) to minimise bias due to experimental effects. There was no significant differences in SMP between controls scanned on different scanners ($t(36) = 1.86$, $p = 0.071$). The demographics of the final cohort are given in Table 1. Between-group analysis of variance for age was non-significant ($F(2,103) = 1.43$, $p = 0.24$) and similarly for chi-squared tests of gender ($\chi^2 = 3.81$, $p = 0.15$).

Two participants with MDD had a comorbid diagnosis of psychosis for which they were prescribed risperidone. These two participants along with 14 others from the MDD group (i.e. 16 in total) were currently administered with selective serotonin reuptake inhibitors. Details are given in S1 Table. There were no significant differences in age ($t(33) = -0.972$, $p=0.36$) or gender ($\chi^2 = 2.29$, $p = 0.13$) between unmedicated and medicated sub-groups of MDD participants.

Comparisons of SMPs derived from correlations between wavelet coefficients at other scales are displayed in S2 and S3 Tables (scale 3, 0.03 - 0.06Hz) and S4 and S5 Tables (scale 4, 0.02 - 0.03Hz).
